# Biodistribution and dosimetry of ^177^Lu-DOTA-IBA for therapy of bone metastases

**DOI:** 10.1186/s13550-024-01094-6

**Published:** 2024-03-22

**Authors:** Hongmei Li, Wenjie Pei, Xiqun Yang, Gengcuo Qu, Qingchu Hua, Lin Liu, Yudi Wang, Tingting Xu, Yue Chen

**Affiliations:** 1 Department of Nuclear Medicine, The Affiliated Hospital of Southwest Medical University, No 25 TaiPing St, Jiangyang District, Luzhou, 646000 Sichuan People’s Republic of China; 2 Nuclear Medicine and Molecular Imaging Key Laboratory of Sichuan Province, Luzhou, 646000 Sichuan People’s Republic of China; 3 Institute of Nuclear Medicine, Southwest Medical University, Luzhou, 646000 Sichuan People’s Republic of China; 4 Department of Dermatology, Affiliated Hospital of Southwest Medical University, Luzhou, 646000 Sichuan People’s Republic of China

**Keywords:** ^177^Lu-DOTA-IBA, Dosimetry, Biodistribution, Bone metastasis, Radionuclide therapy

## Abstract

**Background:**

We designed and synthesized a novel bisphosphonate radiopharmaceutical (^68^ Ga- or ^177^Lu-labeled DOTA-ibandronate [^68^ Ga/^177^Lu-DOTA-IBA]) for the targeted diagnosis and treatment of bone metastases. The biodistribution and internal dosimetry of a single therapeutic dose of ^177^Lu-DOTA-IBA were evaluated using a series of single-photon emission computerized tomography (SPECT) images and blood samples. Five patients with multiple bone metastases were included in this prospective study. After receiving 1110 MBq ^177^Lu-DOTA-IBA, patients underwent whole-body planar, SPECT/CT imaging and venous blood sampling over 7 days. Dosimetric evaluation was performed for the main organs and tumor lesions. Safety was assessed using blood biomarkers.

**Results:**

^177^Lu-DOTA-IBA showed fast uptake, high retention in bone lesions, and rapid clearance from the bloodstream in all patients. In this cohort, the average absorbed doses (ADs) in the bone tumor lesions, kidneys, liver, spleen, red marrow, bladder-wall, and osteogenic cells were 5.740, 0.114, 0.095, 0.121, 0.095, and 0.333 Gy/GBq, respectively. Although no patient reached the predetermined dose thresholds, the red marrow will be the dose-limiting organ. There were no adverse reactions recorded after the administration of 1110 MBq ^177^Lu-DOTA-IBA.

**Conclusion:**

Dosimetric results show that the ADs for critical organs and total body are within the safety limit and with high bone retention. It is a promising radiopharmaceutical alternative for the targeted treatment of bone metastases, controlling its progression, and improving the survival and quality of life of patients with advanced bone metastasis.

**Supplementary Information:**

The online version contains supplementary material available at 10.1186/s13550-024-01094-6.

## Background

The bone is a common metastatic site of malignant tumors. It is associated with complications such as severe intractable pain in two-third of patients, which could be associated with spinal cord compression and pathological fractures [1, 2]. Early diagnosis and treatment of bone metastasis are of great significance to improve the patient's quality of life and prolong survival [3, 4]. The most common forms of palliative care include external irradiation, morphine-derived analgesics and bisphosphonates. β-Emitting, bone-seeking radionuclides control pain in prostate cancer metastatic to bone with pain response rates in the order of 60–70% when used as single agents [5]. ^89^SrCl and ^223^RaCl_2_ were approved by the FDA for the treatment of bone metastases from tumors. These widely used radiopharmaceuticals for bone metastases provide significant relief from bone pain and reduce the incidence of bone-related events [6, 7]. Lutetium-177 (T_1/2_ = 6.73 days, E_βmax_ = 497 keV, E_γ_ = 113 keV [6.4%], 208 keV [11%]) are therapeutic radioactive nuclides for bone pain relief, characterized by relatively low beta particle energy and long physical half-life [8, 9]. A model calculation showed that ^177^Lu is an ideal radionuclide for full beta particle energy deposition in a small tumor volume [10]. Bisphosphonates are widely used as anti-bone resorption agents for bone metastases. Several studies of ^177^Lu-labelled bisphosphonates, such as ^177^Lu-EDTMP and ^177^Lu-BPAMD were investigated for bone metastasis therapy [11–13]. Ibandronate acid is a third bisphosphonate with very high hydroxyapatite affinity and inhibition of the farnesyl diphosphate synthase [14]. The preclinical and first clinical evaluation of combining radionuclides with the anti-bone metastatic drug IBA has high potential to relieve bone pain caused by inoperable multiple bone metastases through intravenous radionuclide therapy [15–17]. Biodistribution and bone uptake of ^68^ Ga- or ^177^Lu-labelled compounds are comparable [15, 17]. ^68^ Ga-/^177^Lu-DOTA-IBA provide a set of potential theranostic radiopharmaceuticals, enabling patient-individual dosimetry and pre- and post-therapeutic evaluation.

This prospective study aimed to evaluate the dosimetry and safety of a single therapeutic activity administration of ^177^Lu-DOTA-IBA in patients with metastatic bone tumors, based on a series of single-photon emission computerized tomography (SPECT/CT) images and blood samples.

## Materials and methods

### Study design and patients

Five patients were enrolled in this study. This study was approved by the Institutional Review Board of the Affiliated Hospital of Southwest Medical University (KY2022114). All the patients (three patients had lung cancer, and two had breast cancer) showed evidence of bone metastasis on ^99m^Tc-MDP bone scans. Patients received surgery, radiotherapy, chemotherapy, endocrine therapy, immunotherapy, targeted therapy, or diphosphonates as previous treatments and were on palliative treatment if no other treatment options were available. Adequate bone marrow function, including a hemoglobin level of over 60 g/L, total leukocyte count greater than 2.5 × 10^9^/L, and platelet count greater than 60 × 10^9^/L was required for eligibility and the life expectancy had to be at least 3 months. The exclusion criteria were the following: (1) a superscan finding on ^99m^Tc-MDP bone scan, (2) presence of pathologic bone fractures or spinal cord compression, (3) age < 18 years, and (4) pregnancy. Written informed consent was obtained from all the patients.

For the enrolled patients, a ^68^ Ga-DOTA-IBA positron emission tomography (PET)/CT scan (after 90 min of intravenous tracer administration) was performed for comparative purposes within 3 days of the ^99m^Tc-MDP bone scan. Blood biomarkers (including routine blood examination, liver function, and kidney function) were evaluated within 3 days before ^177^Lu-DOTA-IBA treatment (baseline) and at 2, 4, and 8 weeks after injection (follow-up).

### ^***177***^***Lu-DOTA-IBA treatment protocol***

The detailed labelling method for ^177^Lu-DOTA-IBA was described in a previous study [16]. In this study, all enrolled patients received radionuclide therapy by means of intravenous injection. Patients were advised to maintain good oral hydration before and after the infusion of ^177^Lu-DOTA-IBA. A total of 1110 MBq of ^177^Lu-DOTA-IBA was administered over 6–10 s followed by a saline flush [18, 19]. Serial ^177^Lu-DOTA-IBA planar whole-body bone scans were performed at 0.5 h, 4 h, 1 day, 3 days, 5 days, and 7 days after ^177^Lu-DOTA-IBA administration. SPECT/CT images of the abdomen were performed at 1 day and 3 days.

### SPECT imaging

All enrolled patients underwent whole-body imaging at six time points after drug administration: 0.5 h (before urination), 4 h, 1 day, 3 days, 5 days and 7 days after injection. Planar acquisitions were performed with a dual-head Symbia SPECT/CT system (Symbia T16, Siemens, Germany) in the supine position using high-energy parallel-hole collimator with a 20% energy window located at the center of the 208 keV photopeak. Continuous acquisition was performed at a scan speed of 10 cm/min and a 1024 × 256 matrix. Three-dimensional SPECT/CT acquisitions were performed with a single field-of-view cantered on the abdomen (including the liver and kidney) using 32 camera steps of 15 s each and an image matrix of 128 × 128 voxels (voxel spacing, 2 mm, the slice thickness, 2 mm).

### Blood and urine sampling

After injection, blood samples were drawn intravenously at 0.5, 4, and 24 h and at various times over 3, 5 and 7 days. The samples were centrifuged in heparinized tubes, with two 1 mL aliquots of plasma were prepared from each blood sample for determination using an automated gamma counter (CAPRAC-t, CAPINTEC, USA).

Urine was collected immediately after infusion of ^177^Lu-DOTA-IBA until approximately 24 h after therapeutic activity administration. All voids were collected in separate plastic bottles, and the volume of urine collected in each bottle was carefully measured. Samples (1 mL) were taken from each plastic bottle and diluted to a total volume of 5 mL before being counted in an automated gamma counter.

### Dosimetric analysis

The following source organs were included for dosimetric calculations: kidneys, red marrow, cortical bone mineral surface, trabecular bone mineral surface, urinary bladder content, and the remainder of the body. Considering that the initial planar image was taken prior to urination following injection, a direct conversion based on the geometric mean counts of the whole-body anteroposterior images during planar imaging, i.e., the counts corresponding to the initial time points corresponded to the administered activity. For SPECT acquisition, the camera calibration factor was determined following one of the approaches proposed by Medical internal radiation dose (MIRD Pamphlet No, 26 [20].). To convert the measured voxel values in the reconstructed SPECT images to ^177^Lu activity, a well-calibrated point source of ^177^Lu (37 MBq) was scanned applying the same acquisition protocol and reconstruction method as used in the patient studies. From this measurement, the calibration factor was determined as 6 cps/MBq. HERMES software (HERMES, Stockholm, Sweden) was used to draw regions of interest (ROIs) and VOIs encompassing the entire source organ or lesion on CT image for calculating organ or lesion volume to determine the percentage of injected activity (%IA) and the normalized radiation-ADs. The percent of injected activity in source organs and lesions was calculated to generate time-activity curves. The parameter was entered into MATLAB (MathWorks, USA) and fitted with a mono- or bi-exponential. The coefficient of determination (R^2^) was calculated by curve fitting procedure in MATLAB to evaluate the correlation. To ensure better correlation data, a function fitting method with an R^2^ closer to 1 was chosen. The %IA for each source organ was entered into OLINDA/EXM version 2.0 model to obtain the residence time.

Disintegrations from the femur regions were used and scaled-up according to the percentage of dry bone weight given by ICRP 70 to estimate the number of disintegrations in the skeletal system owing to the nonuniform uptake of ^177^Lu-DOTA-IBA in the skeleton [21].

DOTA-ZOL is a bisphosphonate that accumulates on the bone mineral surface [22], assuming that DOTA-IBA is also distributed on the bone surface. Cumulative skeletal activity was distributed between the cortical bone mineral surface (80%) and trabecular bone mineral surface (20%) [21].

Red marrow activity uptake was estimated from venous blood samples, and time-activity curves for the red marrow were estimated from venous blood sampling as follows [23]:

$${A}_{redmarrow}(MBq)=\frac{{AC}_{blood}(MBq/mL)\times RMBLR}{1.05\frac{g}{mL}}$$

where, *A* is the activity, AC is the activity concentration, and RMBLR is the red marrow-to-blood activity concentration ratio. Standard values for red marrow mass (1500 g) and density (1.05 g/mL) were used for this estimation. An RMBLR of 1.0 was used as suggested for ^177^Lu-therapy [24]. To test the hypothesis above by measuring the RM effective half-life in VOI placed in lumbar vertebrae obtained from SPECT at 1 and 3 days after therapeutic activity administration (mono-exponential fit), from blood sample at the same time (mono-exponentially fit) and from the second (slow) exponential decay time constant obtained using all the 6 time points.

Urine was collected within 24 h after the administration, and the attenuation correction activity (MBq) of the subjects' excreted urine was input into the Hermes software to supplement the attenuation caused by biological excretion. The bladder voiding interval was 2 h, and the bladder voiding model was used for calculations. The ICRP-103 formalism was used to calculate the AD and ED to the whole body and organs, and the results were divided by the injected activity to obtain the mean AD (mGy/MBq) and mean ED (mSv/MBq) of the patients.

Diagnostic PET/CT images were used to select tumor lesions of interest and to determine the lesion volume, using a threshold of 40% of the SUVmax in the PET images [25]. To reduce the impact of partial volume effect, we don’t select lesions that are too small (≤ 5 cm.^3^). Tumor volumes were individually considered and assumed to have the same mass density as cortical bone (1.92 g/mL) [26]. A sphere model available in the same software was used (OLINDA/EXM version 2.0 (Hermes Medical Solutions, Stockholm, Sweden)) for the AD in the tumor. We adopted the safety dose threshold of 2 Gy and 23 Gy for the red marrow and the kidneys respectively [27, 28]

### Error estimation

The choice of imaging protocol (i.e., planar, SPECT, or hybrid imaging) can affect the measurement of the activity estimates that are the basis for dosimetry calculations. Time-integrated activity coefficients (TIACs) are influenced by the selection model [29, 30]. We selected the Committee on Medical internal radiation dose (MIRD) to obtain the source organ TIACs. The volume or mass of an organ or tumor is usually obtained from the VOI outlined on anatomical or functional imaging data. The segmentation of organs and lesions can also affect the ADs. Any factor that affects VOI will affect AD calculation. However, no error propagation was applied, which weakened the values of the presented results in this study.

### Evaluating side effects and toxicity

General follow-up toxicity and adverse effects were assessed at 2, 4, and 8 weeks after ^177^Lu-DOTA-IBA injection using blood biomarkers, including routine blood examination, liver function, and kidney function. Follow-up results were compared with those at baseline. Toxicities were graded according to the Common Terminology Criteria for Adverse Events, version 5.0 [31].

### Statistical analysis

Blood biomarker data at baseline and 8 weeks after injection were compared using the paired Wilcoxon test. Statistical analysis was performed using SPSS statistical software (version 22.0; SPSS Inc., Chicago, Illinois, USA). Statistical significance was set at *p* < 0.05.

## Results

We enrolled five female patients (mean age of 39 ± 5.5 years, range: 33–62 years), with bone metastases (*n* = 3 from lung cancers, *n* = 2 from breast cancer) to evaluate the safety and dosimetry of a therapeutic activity administration of 1110 MBq of ^177^Lu-DOTA-IBA. Additional patient statistics are reported in Additional file 1: Table S1.

### Biodistribution

**Fig. 1:**
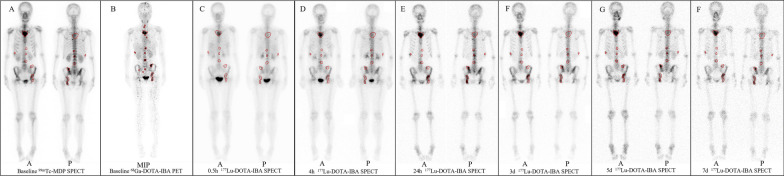
Comparison of ^99m^Tc-MDP with ^68^ Ga-DOTA-IBA in patient, and of whole-body planar images at 0.5 h, 4 h, 24 h, 3 d, 5 d, and 7 d after injection for the representative patient (patient 4) (tumors with red lines)

Representative whole-body planar images at each interval are shown in Fig. 1. The outline of the kidneys and bladder was visible on 0.5 h planar imaging. Strong uptake occurred in the skeletal system from 4 h onwards. Tracer uptake was higher in the kidney, bladder-wall, bone, and bone metastases but lower in the liver and spleen. In subsequent images, there was little specific uptake in the kidneys and soft tissues, with persistent uptake in skeletal lesion. ^177^Lu-DOTA-IBA was mainly cleared through the urinary system.

**Fig. 2:**
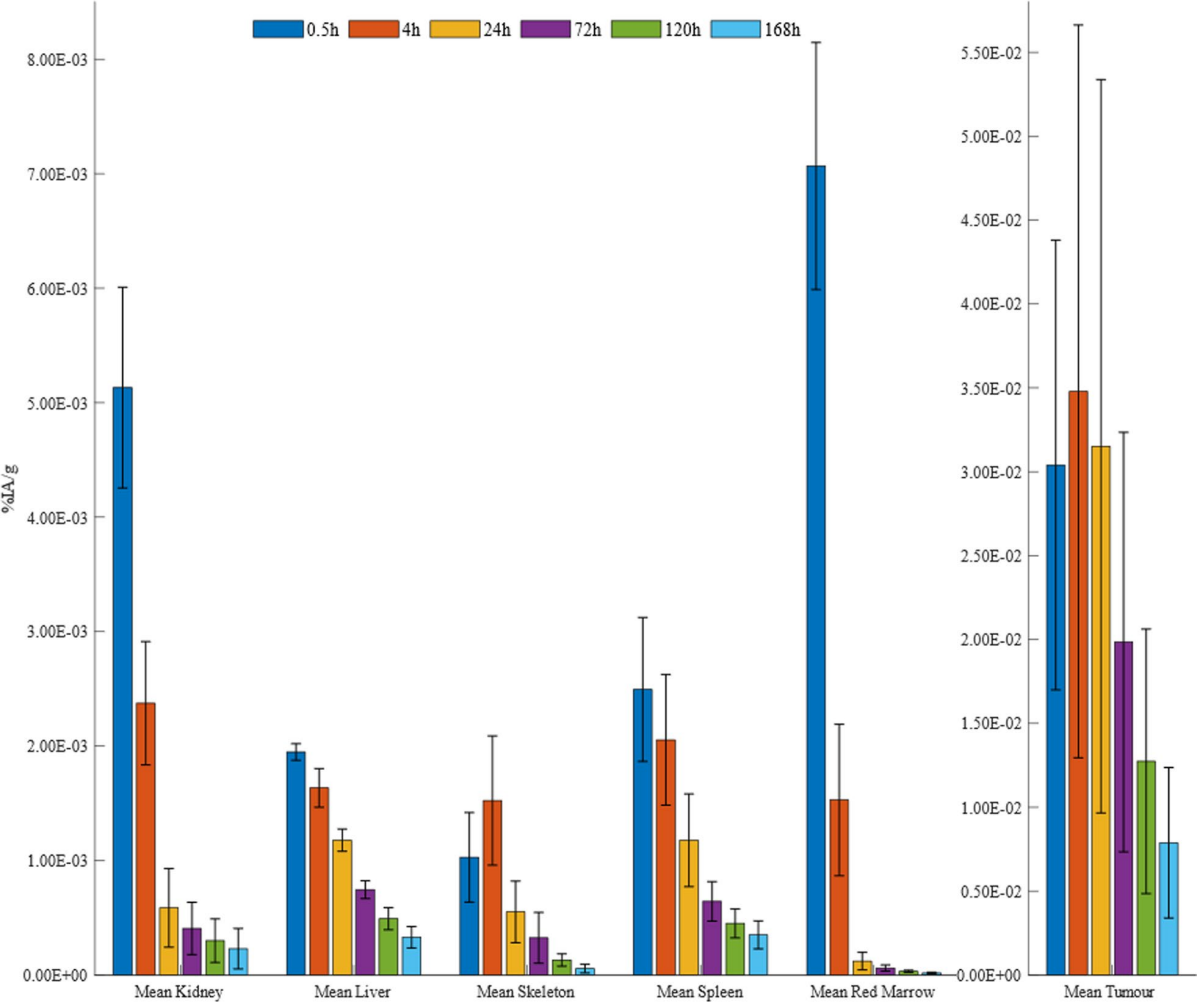
The percentage injected activity (%IA/g) of ^177^Lu-DOTA-IBA in the target organs versus time.

The time-activity histogram plots are expressed as percentage injected activity per gram (%IA/g) and corrected for the physical decay of the radionuclide at the acquisition time (Fig. 2). Red marrow histogram plots showed that the clearance rate in the patient group was fast, with a mean %IA/g of 7.1 × 10^–3^ ± 1.1 × 10^−3^ at 0.5 h after injection and 1.2 × 10^−4^ ± 7.7 × 10^−5^ at 24 h after injection. In contrast, rapid uptake and high retention of ^177^Lu-DOTA-IBA were observed in the bone, with an uptake of 1.5 × 10^–3^ ± 5.6 × 10^−4^%IA/g at 4 h after injection. Even at 72 h after injection, the activity in the skeleton was about 3.2 × 10^−4^ ± 2.2 × 10^−4^%IA/g. High retention of ^177^Lu-DOTA-IBA in tumor lesions was found, averaging 3.5 × 10^−2^ ± 2.2 × 10^−2^%IA/g at 4 h after injection and approximately 3.2 × 10^−5^ ± 9.8 × 10^−6^%IA/g at 120 h after injection. The time-activity histogram plots of the liver, kidneys, and spleen showed low uptake. The time–activity curves are presented in Additional file 1: Figures S1–S2. At 4 h, the systemic %IA was only 59% of the initial value and the bladder contents continued to accumulate, confirming that 41% was metabolized by the kidneys. Additional file 1: Figures S3–S4 show the organs and lesions segmentation along different time points.

### Safety dosimetry

Table 1 shows the AD (mGy/MBq), mean ED (mSv/MBq) and Residence time (MBq/MBq.h) of ^177^Lu-DOTA-IBA. Table 2 details the normalized ADs for the target organs. The mean ED for the whole body is 0.055 ± 0.008 mSv/MBq and 59.94 mSv at an administered active dose of 1110 MBq. The red marrow is the dose-limiting organ and absorbed the dose at 0.095 ± 0.022 Gy/GBq. The osteogenic cells absorbed the highest dose at 0.657 ± 0.127 Gy/GBq. The bladder-wall absorbed the dose at 0.333 ± 0.050 Gy/GBq. The kidney is a major metabolic organ, and absorbed a dose of 0.114 Gy/GBq, followed by the bladder-wall (0.333 ± 0.050 Gy/GBq), spleen (0.121 ± 0.035 Gy/GBq), and liver (0.095 ± 0.051 Gy/GBq). The kidneys showed a much lower normalized AD than the red marrow and osteogenic cells. Additional file 1: Table S2 shows the biological half-life in source organs. Biological half-life: kidney 111.89 ± 38.82 h, skeleton 65.15 ± 7.40 h.

**Table 1:** AD (mGy/MBq), mean ED (mSv/MBq) and Residence time (MBq/MBq.h) of ^177^Lu-DOTA-IBA

Organs	AD		Residence time	
	Mean (*n* = 5)	± SD	Mean (*n* = 5)	± SD
Adrenals	0.030	0.005	-	-
Brain	0.026	0.005	-	-
Breasts	0.025	0.004	-	-
Esophagus	0.026	0.005	-	-
Eyes	0.026	0.005	-	-
Gall bladder wall	0.027	0.005	-	-
Left colon	0.027	0.005	-	-
Small Intestine	0.027	0.005	-	-
Stomach wall	0.027	0.005	-	-
Right colon	0.027	0.005	-	-
Rectum	0.029	0.005	-	-
Heart wall	0.026	0.005	-	-
Kidneys	0.114	0.022	0.354	0.069
Liver	0.095	0.051	1.462	0.803
Lungs	0.026	0.005	-	-
Ovaries	0.028	0.005	-	-
Pancreas	0.028	0.005	-	-
Salivary glands	0.025	0.004	-	-
Red marrow	0.095	0.022	0.482	0.19
Osteogenic cells/skeleton	0.657	0.127	12.35	2.394
Spleen	0.121	0.035	0.179	0.053
Thymu	0.026	0.005	-	-
Thyroid	0.026	0.004	-	-
Urinary bladder wall	0.333	0.050	1.139	0.182
Uterus	0.029	0.005	-	-
Total body/reminder	0.049	0.008	15.854	2.808
Effective dose	0.055	0.008	-	-

**Table 2:** The normalized ADs for the target organs (mGy/MBq)

Organ	Patient no					Mean (*n* = 5)	SD
	1	2	3	4	5		
Liver	0.169	0.091	0.132	0.045	0.036	0.095	0.051
Kidneys	0.145	0.112	0.078	0.110	0.127	0.114	0.022
Spleen	0.131	0.168	0.127	0.119	0.061	0.121	0.035
Red marrow	0.121	0.080	0.069	0.084	0.120	0.095	0.022
Bladder	0.317	0.298	0.318	0.302	0.431	0.333	0.050
Osteogenic cells	0.826	0.570	0.487	0.621	0.779	0.657	0.127
Effective dose	0.068	0.050	0.050	0.046	0.061	0.055	0.008
Maximum tolerated injected activity (GBq)	16.5	25.2	28.9	23.8	16.7	22.2	4.9

The red marrow was the dose-limiting organ for all patients assuming maximum tolerated doses of 2 and 23 Gy for the red marrow and kidneys, respectively. The maximum safe injectable activity (i.e., activity leading to a dose that did not surpass any of the defined maximum tolerated doses) ranged from 16.5 to 28.9 GBq.

### Tumor dosimetry

Tumor masses were determined from segmented lesion volumes performed on the tomographic images, assuming a density of 1.92 g/ml. The absorbed doses for the tumor lesions are displayed in Table 3. The absorbed doses for the tumor lesions ranged from 2.82 to 9.50 Gy/GBq. The mean absorbed tumor dose per patient ranged from 3.87 to 6.96 Gy/GBq. Differences within groups and individuals are acceptable owing to differences in the location, size, and nature of the lesions (Additional file 1: Table S3).

**Table 3:** The absorbed doses for the tumor lesions(mGy/MBq)

Lesion	Patient no				
	1	2	3	4	5
1	4.33	2.84	6.20	7.49	4.68
2	6.80	5.63	9.50	8.20	5.22
3	6.20	2.82	6.11	9.01	3.07
4	6.68	5.06	6.80	5.79	7.48
5	4.01	2.97	4.25	4.32	5.40
Mean	5.60	3.87	6.57	6.96	5.17
SD	1.19	1.22	1.70	1.69	1.41

Overall tumor statistics were a mean of 5.74, and an SD of 1.73 (*n* = 25)

### Evaluating safety and adverse events

**Fig. 3:**
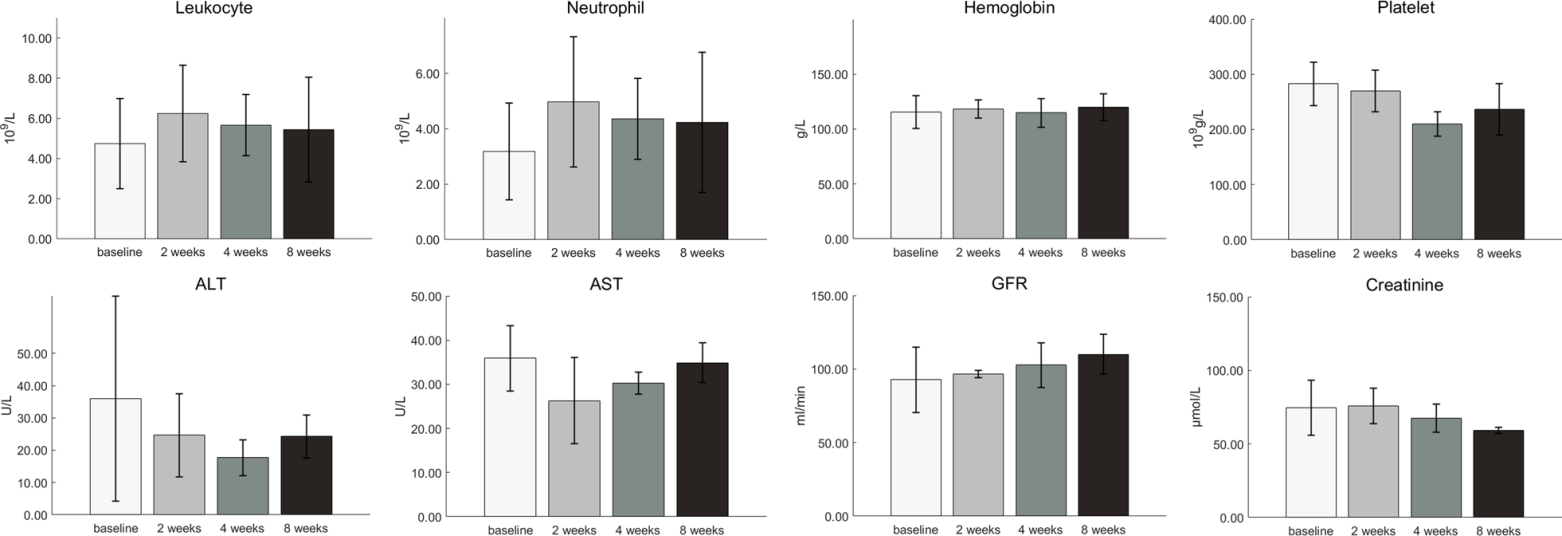
Selected biomarkers at baseline, 2 weeks, 4 weeks, and 8 weeks after ^177^Lu-DOTA-IBA therapy (*ALT* Alanine aminotransferase, *AST* Aspartate aminotransferase, *GFR* Glomerular filtration rate)

All patients tolerated ^177^Lu-DOTA-IBA treatment well, and ^177^Lu-DOTA-IBA had no statistically significant effect on bone marrow hematopoiesis (white blood cells, neutrophils, hemoglobin, and platelets), liver function (ALT and AST), or renal function (GFR, creatinine) (Fig. 3, *P* > 0.05). There was a significant decrease in platelets at 4 weeks after injection, which was temporary and recovered by 8 weeks.

## Discussion

Ibandronic-acid presents as an ideal candidate for labeling with the therapeutic radionuclide lutetium-177 for radionuclide therapy of bone metastases, as it shows high osteoclast and hydroxyl apatite binding [16]. Preclinical small animal studies using ^177^Lu-DOTA-IBA and ^68^ Ga-DOTA-IBA showed comparable results, suggesting that the two tracers are new theranostic pairs for bone-targeted radionuclide therapy [15–17]. In this study, dosimetry, biodistribution, and safety evaluations of ^177^Lu-DOTA-IBA were performed for five patients with bone metastases. Radiopharmaceuticals can be safely used to relieve bone pain in patients with metastatic bone injuries [15]. The source organs identified for dose analysis included the liver, kidneys, spleen, red marrow, bladder, skeleton, and whole body. The biphasic kinetic behavior of ^177^Lu-DOTA-IBA was observed in all source organs and throughout the body. Double exponential curve fitting was used to calculate the time-activity curve (fit constant (R^2^), Additional file 1: Table S4). For all patients, the red marrow is the potential dose-limiting organ that allows for a maximum administered activity of 16.5–28.9 GBq. Overall, the low dose administered activity of 1.11 GBq resulted in a much lower dose of red marrow than the defined dose limit of 2 Gy for red marrow. ^177^Lu-DOTA-IBA has emerged as a bone-seeking agent that is more specific for bone metastasis and has a lower burden on red marrow and normal bone in the skeleton than other radiotherapeutic compounds. The studied level of ^177^Lu-DOTA-IBA administered activity was safe. In subsequent studies, the patient's therapeutic administered activity can be increased to explore the relationship of dose–response.

In this study, a conservative RMBLR value of 1.0 was applied as suggested for ^177^Lu-based peptide receptor radionuclide therapy [32]. The use of different dose calculators or assumptions may lead to varying results. To verify this hypothesis, the RM effective half-life was measured by three methods (Additional file 1: Table S5). That obtained from SPECT imaging (VOI placed in lumbar vertebrae at 1 and 3 days, mono-exponential fit), blood sample at the same time (mono-exponential fit), all imaging of the six time points (the second (slow) exponential decay time). The difference in calculated half-lives between the two protocols in P1 may be attributed to the presence of more metastatic lesions in the lumbar spine. During lumbar spine VOI outlining, we had to avoid the bone lesions and outline only the remaining lumbar vertebrae without lesions. This resulted in an equal reduction in the percentage of red bone marrow in the remaining lumbar vertebrae, which could have led to some errors. The inconsistent results of the image-based and blood-based protocols in P1 may be related to the reasons mentioned above. Similarly, the inconsistent results of red bone marrow based on blood-based protocols may also be related to the same reasons. The blood-based protocol was chosen for red bone marrow activity estimation primarily because many patients with advanced tumour bone metastases are likely to have metastases in the lumbar spine area. However, our study has a limitation at present. It was conducted based on the absence of specific tracer uptake in red bone marrow, which cannot yet be demonstrated due to the small number of cases. This issue needs to be addressed in future studies.

In this study, tumor uptake was excluded from general bone uptake; however, bone lesions may also lead to a red marrow dose, depending on their location, which may have been underestimated in this study. The results provided in this article are based on calculations using the widely accepted OLINDA/EXM version 2.0, which makes specific assumptions for bone marrow dose calculation [32]. Therefore, activity is possibly distributed on the surface of bones, and the skeleton cumulated activity is distributed between the cortical bone mineral surface (80%) and the trabecular bone mineral surface (20%).

The limiting organs for the ^177^Lu peptide receptor therapy are red marrow and kidneys. The kidneys were not restricted owing to its low intake and rapid clearance. The maximum tolerable injection activity ranged from 158.6 to 294.9 GBq when the renal AD threshold was 23 Gy. The average AD to the bladder-wall was 0.333 Gy. The AD of red marrow and kidney is 494% (mean value of 0.47 versus 0.095 Gy/GBq) and 491% (mean value of 0.56 versus 0.114 Gy/GBq), which was higher than the findings of this study. Differences in the AD of red marrow can be expected because of different assumptions in the calculation of red marrow. More importantly, the current study combines three-dimensional SPECT imaging, whereas Qiu et al.'s AD assessment was solely based on planar imaging. The combination of three-dimensional SPECT and planar dosimetry has the advantage of accurately segmenting interested organs and structures, reducing activity from overlapping structures. Significant differences were observed in the tumor doses ranging from 2.82 to 9.50 Gy/GBq from the data of the 25 tumor lesions (Table 3). These changes in bone lesions may be attributed to different osteoblast activities during bone injury.

^177^Lu-DOTA-IBA was directly compared to other therapeutic radiopharmaceuticals used for bone remission (Table 4) [33–38]. Although a direct comparison of multiple radiopharmaceuticals is challenging owing to the different dosimetry methods, ^177^Lu-DOTA-IBA may have more favorable treatment indicators (red marrow AD and the tumor-to-RM AD ratio) than ^177^Lu-DOTA-ZOL. Furthermore, ^177^Lu-DOTA-IBA showed the lowest red marrow dose and the highest tumor-to-RM dose ratio. Therefore, ^177^Lu-DOTA-IBA may cause fewer bone-related side effects than similar therapies. Although a dose of 1.11 GBq is very safe, the treatment of ^177^Lu-DOTA-IBA should be carefully planned, and personalized monitoring should be carried out in terms of injection activity and number of cycles to achieve optimal efficacy and avoid serious side effects.

**Table 4:** Comparison the AD (mGy/MBq) of ^177^Lu-DOTA-IBA to other radiopharmaceuticals used for bone metastasis treatment

Parameter	^177^Lu-DOTA-IBA	^177^Lu-DOTA-ZOL [33]	^89^SrCl_2_ [37]	^153^Sm-EDTMP [38]	^177^Lu-EDTMP [38]	^188^Re-HEDP [35]	^223^RaCl_2_
Tumor lesion	5.74 ± 1.73	4.21 ± 2.40	233 ± 166	6.22 ± 4.21	6.92 ± 3.92	3.83 ± 2.01	179.8(68–490) [36]
Red marrow	0.095 ± 0.02	0.36 ± 0.12	18.9	1.41 ± 0.6	0.83 ± 0.21	0.61 ± 0.21	73.9[34]
Tumor–to–red marrow dose ratio	60.42	13.9	12.3	4.4	8.31	6.28	2.4

## Conclusion

^177^Lu-DOTA-IBA is a novel radiopharmaceutical with promising pharmacokinetics for treating bone metastases. This study evaluated the safety and dosimetry of a single therapeutic dose of ^177^Lu-DOTA-IBA, which showed high uptake and residence time in the bone lesions. ^177^Lu-DOTA-IBA is safe, and an promising agent in the treatment of metastatic bone pain. The obtained results, compared to those of established bone-targeting agents, underline the clinical potential and possible benefits of ^177^Lu-DOTA-IBA for therapy in patients with cancers that metastasize into the bones.

### Supplementary Information

**Additional file 1:** Supplemental material.

## Data Availability

The datasets and materials during the present study are available from the corresponding author on reasonable request.

## Abbreviations

^68^ Ga: Gallium 68
^177^Lu: Lutetium-177
DOTA-IBA: DOTA-ibandronate
AD: Absorbed dose
ED: Effective dose
SPECT: Single-photon emission computerized tomography
CT: Computed tomography
VOI: Volume of interest
%IA/g: Percentage injected activity per gram
%IA: Percentage injected activity
ROI: Region of interest
RMBLR: Red marrow-to-blood activity concentration ratio
PET: Positron emission tomography
DOTA-ZOL: DOTA- zoledronic acid
MIRD: Medical internal radiation dose
