# Supplementary material for: Biodistribution and dosimetry of 177Lu-DOTA-IBA for therapy of bone metastases
Source: EJNMMI Res. 2024 Mar 22;14:30. doi: 10.1186/s13550-024-01094-6 (PMC10959900; doi:10.1186/s13550-024-01094-6)
Supplement: Supplementary file 1 — Additional file 1: Supplemental material. [file 13550_2024_1094_MOESM1_ESM.docx]

Supplementary Table 1: The basic characteristics of 5 patients.

| P.ID | Age  (Years) | Weight  (kg) | Activity administered  (MBq) | Diagnosis |
| --- | --- | --- | --- | --- |
| P1 | 62 | 65 | 1195.47 | Adenocarcinoma of the lung |
| P2 | 33 | 49 | 1204.72 | Breast adenocarcinoma |
| P3 | 45 | 47 | 1135.53 | Adenocarcinoma of the lung |
| P4 | 34 | 42 | 1099.27 | Breast adenocarcinoma |
| P5 | 44 | 54 | 1088.17 | Adenocarcinoma of the lung |

Supplementary Table 2. The biological half-life (h) in source organs.

| Organ | Patient no. | | | | | Mean | SD |
| --- | --- | --- | --- | --- | --- | --- | --- |
|  | 1 | 2 | 3 | 4 | 5 |  |  |
| Kidney | 69.05 | 77.38 | 163.21 | 128.76 | 121.03 | 111.89 | 38.82 |
| Liver | 70.53 | 115.39 | 130.93 | 115.39 | 66.41 | 99.73 | 29.27 |
| Skeleton | 57.34 | 47.16 | 77.63 | 41.15 | 57.12 | 56.08 | 13.87 |
| Spleen | 54.12 | 64.01 | 71.99 | 63.66 | 71.99 | 65.15 | 7.40 |
| Red Marrow | 4.80 | 5.01 | 6.77 | 1.64 | 1.52 | 3.95 | 2.29 |

Supplementary Table 3. Lesions selected to calculation ADs.

| Patient ID. | location | Nature | Volume(cm^3^) |
| --- | --- | --- | --- |
| P1 | sternum | mixed | 9.3 |
|  | T8 centrum | mixed | 9.5 |
|  | T10 centrum | osteolytic | 8.5 |
|  | L5 centrum | mixed | 7.8 |
|  | Right iliac bone | osteolytic | 14.3 |
| P2 | T12 centrum | mixed | 19.1 |
|  | L1 centrum | osteolytic | 12.3 |
|  | L2 centrum | osteolytic | 9.38 |
|  | L4 centrum | osteolytic | 16.3 |
|  | sacrum | mixed | 17.4 |
| P3 | T10 centrum | osteogenic | 8.5 |
|  | T11 centrum | osteogenic | 7.8 |
|  | L2 centrum | osteogenic | 22.9 |
|  | sacrum | osteogenic | 61.5 |
|  | Right iliac bone | osteogenic | 25.5 |
| P4 | sternum | osteogenic | 22.5 |
|  | T12 centrum | osteogenic | 7.18 |
|  | L4 centrum | osteogenic | 11.7 |
|  | Left iliac bone | osteogenic | 15.7 |
|  | accetabulum | osteogenic | 31.9 |
| P5 | T3 centrum | mixed | 11.8 |
|  | L2 centrum | mixed | 17.1 |
|  | L4 centrum | mixed | 33.2 |
|  | Left iliac bone | mixed | 63.1 |
|  | sacrum | mixed | 52.9 |

|  | Mean（n=5） | SD |
| --- | --- | --- |
| Left Kidney | 0.976 | 0.024 |
| Right Kidney | 0.988 | 0.009 |
| Liver | 0.983 | 0.030 |
| Red Marrow | 0.916 | 0.094 |
| Spleen | 0.965 | 0.022 |
| Lesion | 0.990 | 0.013 |

Supplementary Table 4. Fit constant (R^2^) for the source organs and lesion.

| P.ID | RM half-life calculated based on three methods | | |
| --- | --- | --- | --- |
|  | all points imaging  (h) | SPECT imaging  (h) | blood sample  (h) |
| P1 | 36.9 | 58.8 | 38.9 |
| P2 | 130.8 | 73.7 | 114.1 |
| P3 | 66.6 | 95.5 | 80.7 |
| P4 | 55.4 | 47.7 | 42.7 |
| P5 | 72.7 | 70.4 | 47.9 |

Supplementary Table 5. The RM effective half-life by three methods.

Supplementary Figure 1. The percentage injected activity (IA%) of ^177^Lu-DOTA-IBA in the total body and UB contents.


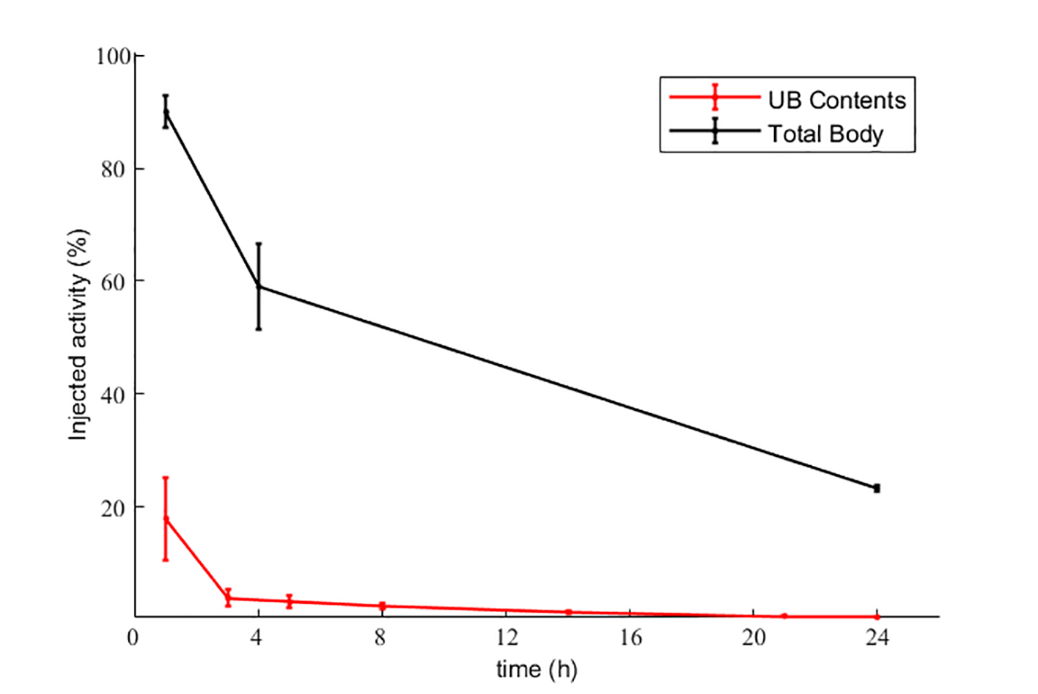


Supplementary Figure 2. The percentage injected activity (IA%) of ^177^Lu-DOTA-IBA in the cortical organs versus time.


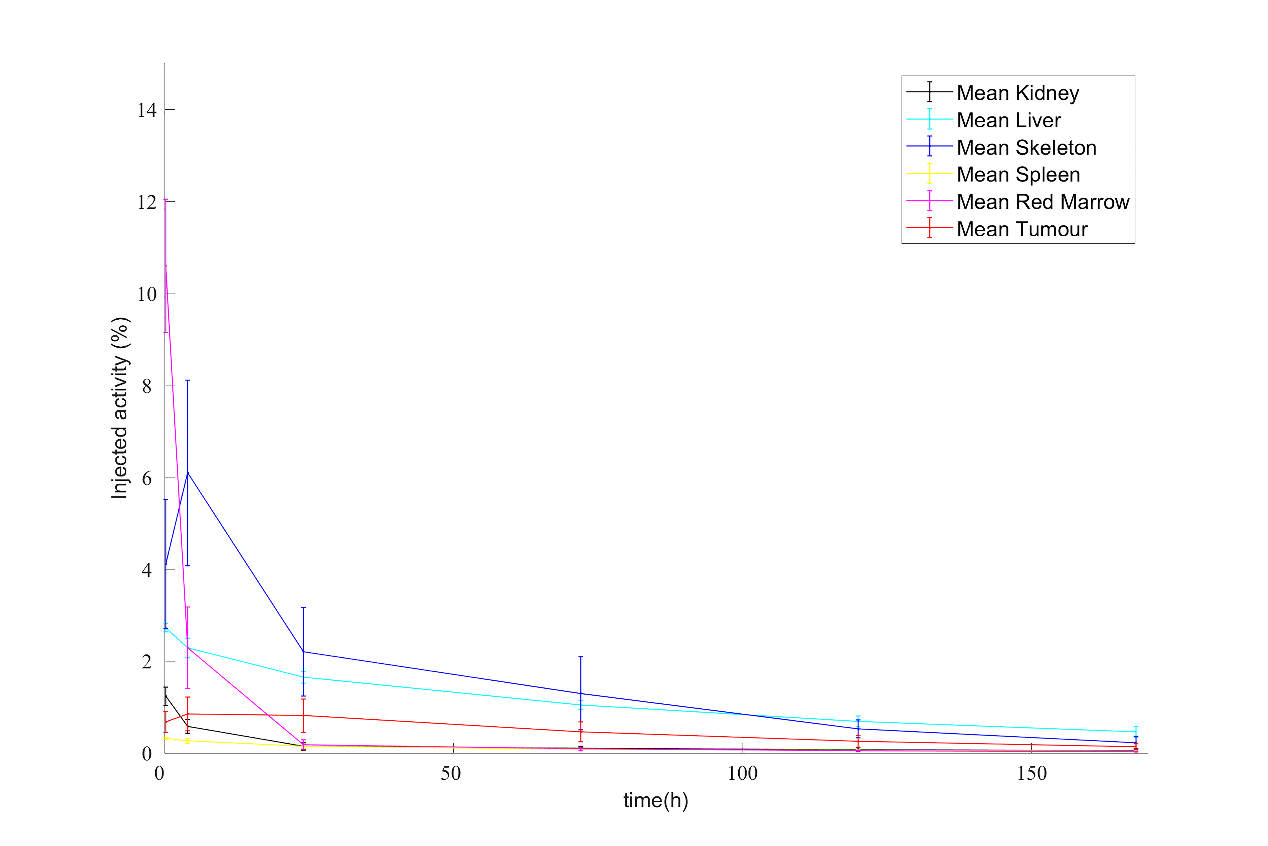


Supplementary Figure 3. Organ segmentation and volume of interest propagation along different time points (patient ID: P1).


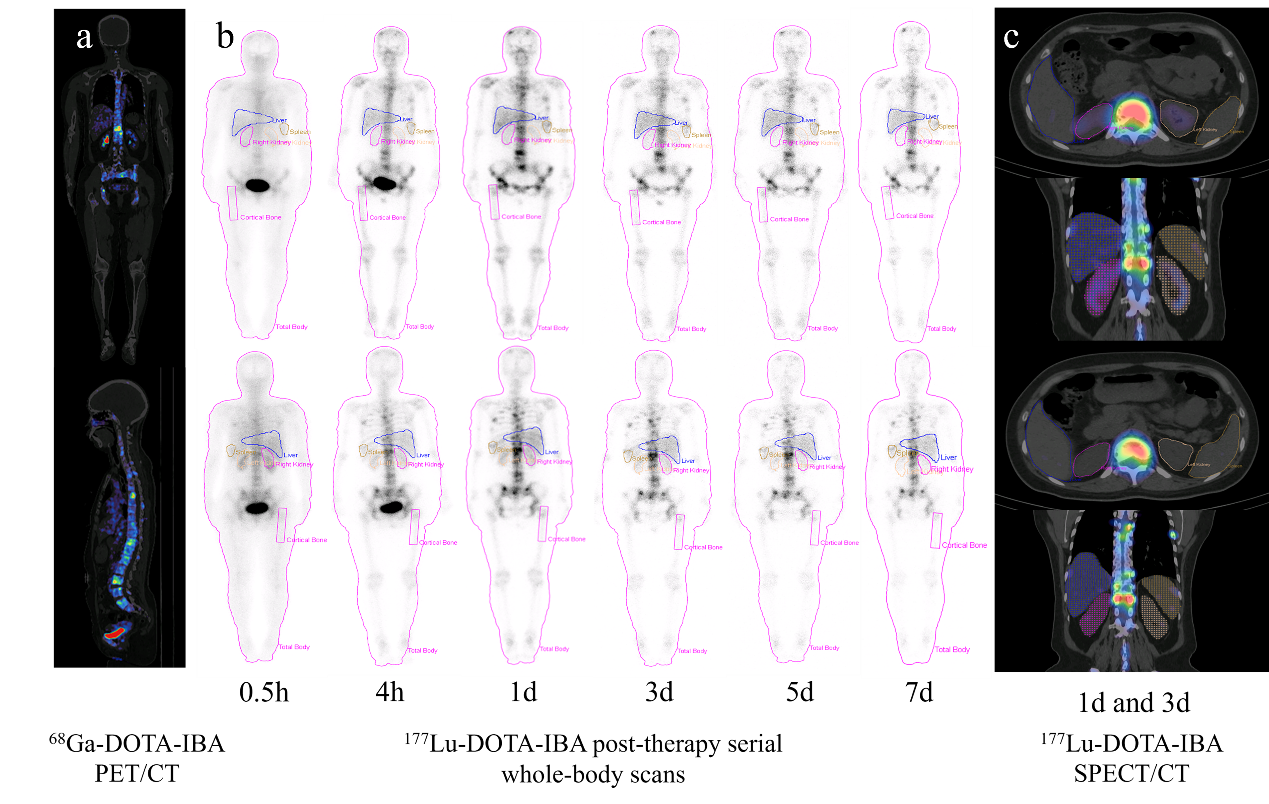


(a).^68^Ga-DOTA-IBA PET/CT images. (b).Liver, kidneys and spleen were delineated. (c).^177^Lu-DOTA-IBA SPECT/CT images show source organs (liver, kidneys, spleen).

Supplementary Figure 4. Lesions segmentation and volume of interest propagation along different time points (patient ID: P1).


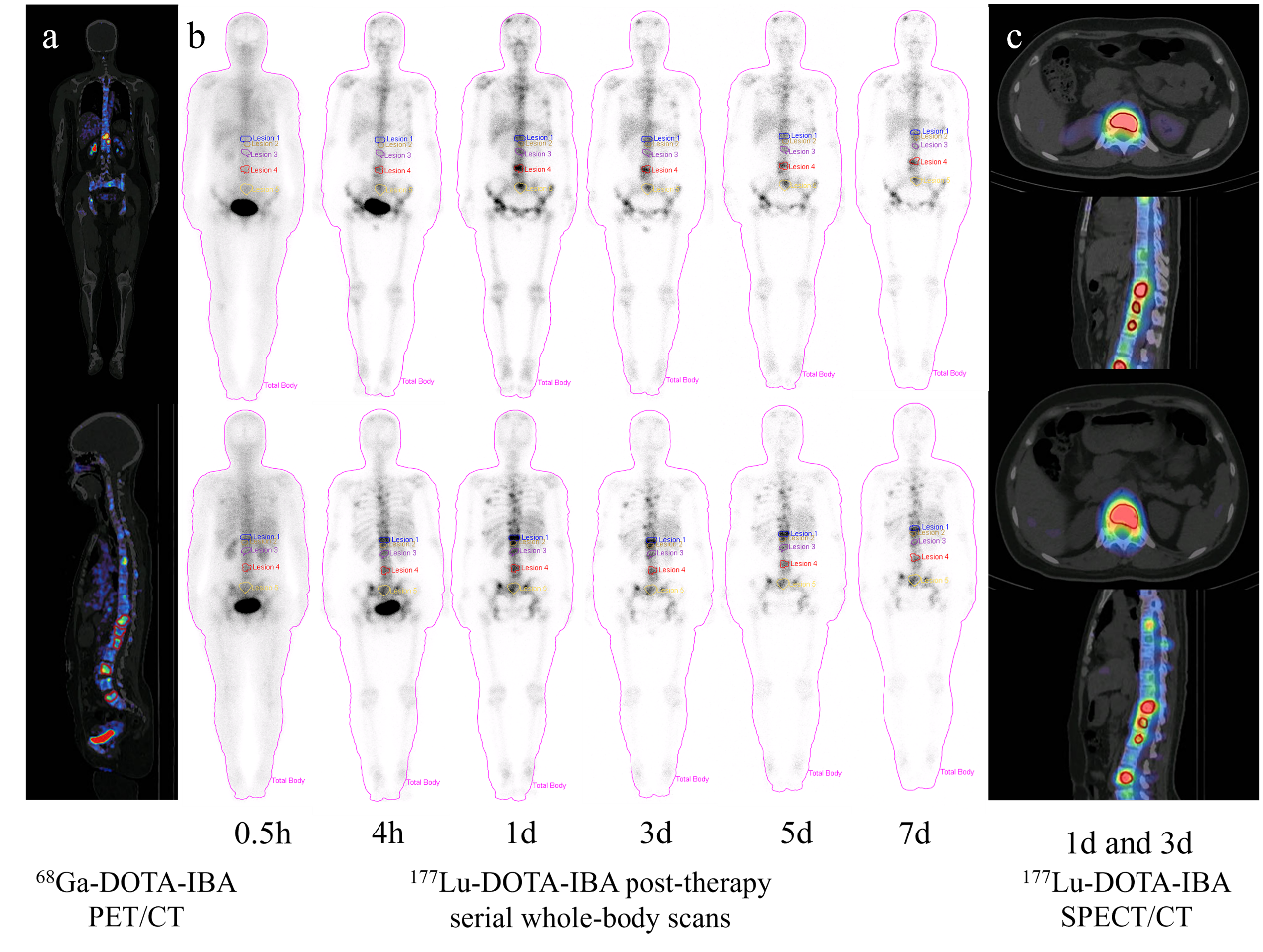


(a).^68^Ga-DOTA-IBA PET/CT images show metastatic lesions on T12, L1, L2, and L4 vertebrae. (b). Serial ^177^Lu-DOTA-IBA whole-body scans images after intravenous injection of 1110 MBq of radiotracer show radiotracer retention (ROIs) in the metastatic sites until 168 h delayed images. (c). ^177^Lu-DOTA-IBA SPECT/CT images show bone metastatic lesions in the vertebrae.
